# Progress in Surgery

**Published:** 1902-02-01

**Authors:** 


### PROGRESS IN SURGERY.

### OTOLOGY.

**Acute Otitis Media.**—H. J. Curtis[1] has recorded a case of primary acute hæmorrhagic otitis media. The affection is an uncommon one. The patient, a hospital nurse, aged 35, was in apparently excellent health after a holiday when the trouble began. She had not been exposed to cold and had never had ear trouble before. First she began to suffer from noises, like bell-ringing, which were referred to the back of the head. About two hours later, when she went to bed, the noises ceased, but excruciating pain came on, which was felt in exactly the same places where the noises had previously been. About four hours later she had the feeling as if something had burst inside the head to the left of the occiput. There was no pain in the ears with this. Then suddenly there was a rapid flow of blood from the left ear, bright at first, then darker. Two handkerchiefs were used to stanch the bleeding, which continued from 2 to 11 A.M., though by the latter time only pale serum escaped. Sixteen days later some impairment of hearing was found by the watch test, but conversation was well heard. There was a large perforation of the left membrana tympani, which was almost occluded by dark blood clot. Both right and left membranes were over-cupped. In addition, there was some hypertrophy of both inferior turbinates and chronic nasopharyngitis. The urine was normal. No discharge occurred after the first attack. Gradually under treatment the perforation closed and hearing became normal. Curtis remarks that the initial symptoms naturally gave rise to some anxiety as to whether there was grave intra-cranial mischief, but that the course of the case soon dispelled this. The naso-pharyngeal condition explains the origin of the trouble, which had evidently followed long-standing Eustachian catarrh. McBride suggests in explanation of these cases that the Eustachian catarrh produces vascular engorgement and then rupture of the vessels in the tympanic mucous membrane, and that the hæmorrhage cuts short the inflammatory process before suppuration occurs. L. Katz[2] has shown that the conical protuberance on the membrane tympani, marking the perforation in some cases of acute otitis media, consists of densely infiltrated granulation tissue covered with highly inflamed epidermis, and springs from the mucosa of the tympanic surface of the membrana tympani. Through the long axis of the cone runs a narrow canal lined with a layer of epithelium which has been invaginated into the perforation. The causes of this formation are a narrow, high-placed perforation, thick exudation, and the presence of a resistant inflamed cutis layer on the membrane. The circumscribed granulation area on the membrane, which is associated with destruction of the membrana propria, is pushed outward by the exudation like a hernia, though it is still covered by the resistant epidermis. The formation of such a cone suggests retention of pus. As soon as observed it should be cut open and dilated, as shown by Schwartze. If this fails to give relief, the cone must be seized with a suitable instrument and removed as close to the membrane as possible. Courtade,[3] writing on the subject of the acute otitis media which occurs in the course of an acute coryza, dismisses the usual routine treatment and lays special emphasis on the sedative and curative effects of insufflation of air. If this treatment is adopted on the appearance of pain, he says the patient is relieved, the head becomes less stuffy, and the deafness diminishes or disappears. At a later stage coarse mucous râles can be perceived with a diagnostic tube. In most cases, especially in children, one insufflation is sufficient to relieve the pain and arrest the progress of the inflammation. The less recent the disease, the less immediate is the relief.

**Chronic Catarrhal Otitis.**—C. M. Cobb, of Boston, Mass.,[4] has written a paper with the title, "Can Nasal-Catarrh and Catarrhal Deafness be Cured?" Much of the treatment for these affections, he says, is palliative and not sufficient to cure. He cites the ordinary history of a case of chronic middle-ear catarrh, which shows that one attack of acute inflammation follows another at varying intervals with partial recovery between the attacks, but with the hearing capacity reduced a little after each until finally it is practically lost. The mechanical devices used to overcome the results of the inflammation by moving the drum membrane and the ossicles do not in any way prevent the acute attacks of inflammation. The ear inflammation has its origin in the naso-pharynx, and reaches the ear either by the sudden blowing of the discharge into the ear through the Eustachian tube, or by the extension of the inflammation along the mucous membrane of the tube. Exceptionally the ear inflammation arises coincidently with that of the upper respiratory tract in the course of some acute diseases. Thus in most cases the chronic catarrh of the ear is a part of the general inflammatory condition of the naso-pharynx. Cobb then proceeds to argue that this condition of the naso-pharynx, except when caused by local syphilitic or tuberculous lesions or by adenoids, is a secondary disease caused by a collection of fluid in one or more of the nasal accessory sinuses, and that it should be treated by improving the drainage of the cavity from which the discharge flows. A chronic discharge, it is pointed out, cannot be cured by washing the surface over which the discharge flows with antiseptic lotions, yet this comprises the usual treatment in nasal and naso-pharyngeal catarrhs. Enlarged lymph follicles in the throat are due to infection and not to bizarre conditions, such as indigestion or uterine disease. The writer then states that he believes it possible in practically all cases to remove the cause of the enlargement of the lymphatic tissue in post-nasal catarrh. If this is accomplished the progress of the deafness will be stopped, and the hearing will improve with the disappearance of the inflammatory thickening in the tympanum. Nasal obstruction *per se* is not considered to be the cause of aural catarrh. For the successful treatment of naso-pharyngeal catarrh the source of the causal discharge must be found. The amount of discharge is not an index of the effect produced, and disastrous results may follow where there is a very slight secretion. A number of cases of ear catarrh and of other affections are then cited, in which a cure resulted from removal of enough of the middle turbinal bone to give good drainage from the accessory sinuses. H. A. Alderton[5] states that the recognition of the mucous type of exudative, non-perforative otitis media is often more difficult than that of the serous type. The affection follows a severe cold. There may or may not be pain. Often intense tinnitus is present. There is a full feeling in the head, hardness of hearing, and possibly dizziness. Iodide of potassium is said to be beneficial, but by far the best treatment is incision and evacuation of the tympanum, repeated if necessary, and always accompanied by regular politzerisation and conscientious treatment of the naso-pharyngeal condition.

[1] Lancet, Aug. 17, 1901, p. 439. [2] Vide abst. Amer. Jour. Med. Sc., Sept. 1901, p. 370. [3] Vide abst. Jour. L. R. and O., July 1901, p. 374. [4] Med. Rec., Sept 7, 1901, p. 371. [5] Vide abst. Med. Rec., Sept. 28, 1901, p. 503.

